# Linear Displacement Calibration System Integrated with a Novel Auto-Alignment Module for Optical Axes †

**DOI:** 10.3390/s20092462

**Published:** 2020-04-26

**Authors:** Yi-Chieh Shih, Pi-Cheng Tung, Yung-Cheng Wang, Lih-Horng Shyu, Eberhard Manske

**Affiliations:** 1 Department of Mechanical Engineering, National Central University, Taoyuan 320, Taiwan; 2 Department of Mechanical Engineering, National Yunlin University of Science and Technology, Yunlin 640, Taiwan; 3 Department of Electro-Optical Engineering, National Formosa University, Yunlin 632, Taiwan; 4 Faculty of Mechanical Engineering, Ilmenau University of Technology, 98693 Ilmenau, Germany

**Keywords:** Fabry–Pérot interferometer, auto-alignment of optical axes, linear displacement calibration

## Abstract

The quality of processed workpieces is affected directly by the precision of the linear stage. Therefore, the linear displacement calibration of machine tools must be implemented before delivery and after employment for a period of time. How to perform a precise calibration with high inspection efficiency is a critical issue in the precision mechanical engineering industry. In this study, the self-developed system integrated by the measurement module based on the common path Fabry–Pérot interferometer for linear displacement and the auto-alignment module for optical axes was proposed to realize the automatic linear displacement calibration of the linear stages. The measurement performance of the developed structure was verified experimentally. With the auto-alignment module, the cosine error was reduced to 0.36 nm and the entire procedure accomplished within 75 s without the limitation of the perceived resolution of the human eye, operational experience, and the risk of misalignment and broken cable. According to the comparison of experimental results for the linear displacement, the repeatability of the proposed measurement module was less than 0.171 μm. After the compensation procedure according to the linear displacement calibration, the systematic positional deviation, repeatability, and accuracy of the linear axis could be improved to 4 μm, 1 μm, and 5 μm respectively. Hence, the calibration efficiency can be improved by 80% with the proposed compact system, which is beneficial for the linear displacement calibration of machine tools in the precision mechanical engineering industry.

## 1. Introduction

Owing to provision of direct traceability for the definition of the meter, laser interferometers have become one of the most major apparatus in the precision mechanical engineering industry while further measuring characteristics, including high resolution, contactless measurement, and capability for various measuring ranges, can be met simultaneously [1,2,3,4,5,6,7,8]. In this section, there are several issues to discuss, including optical alignment before measurement procedure, optical design, and the installation of laser interferometers.

The alignment between optical axes of the laser interferometer and the movement axis of the linear stage is a critical procedure. The purpose is to be capable of performing linear displacement measurement over the whole measuring range. If misalignment or poor alignment quality occurs, a cosine error will be induced and then the measurement accuracy will also be reduced. For this reason, the essential point of optical alignment consists of the operational mode and the available sensing range being performed in fine alignment. For the common commercial laser interferometer system e.g., XL-80 laser measurement system, Keysight Technology 5530 Dynamic Calibrator and API XD LASER [9,10,11], one alignment target is utilized to implement optical alignment by a manual operation. The laser spot incident on this target should be located at the same position either at the start-point or at the endpoint of the whole measuring range. However, the alignment accuracy is limited by the perceived resolution of the human eye. In the newer high-cost interferometer product (Renishaw XM- 60 multi-axis calibrator), a visual interface is introduced to implement fine alignment [12]. Although the operating convenience can be improved, the available sensing range is only within ± 0.25 mm, which means that quite accurate coarse alignment is required before carrying out fine alignment. Furthermore, the entire process is manually operated and the accuracy and the efficiency of this alignment still depends on operational experience. Then in the correlated study [13,14,15], detectors e.g., a position-sensitive detector (PSD) and four-quadrant photodiode detectors placed in the linear stage, are employed as an alignment reference for optical axes. Nevertheless, one detector contains multi-signal and power cables and the corresponding cable should be connected with the power supply, the data acquisition board, or the signal processing module over the whole scale. Therefore, the detector and the corresponding cables will all be pulled together regardless of aligning in any dynamic range, meaning the risk of misalignment and a broken cable may be likely to occur. Once the above-mentioned problem occurs, the alignment procedure should be carried out again, which means that the alignment efficiency will be greatly reduced. In summary, the conventional alignment method is limited by the perceived resolution of the human eye and the problem of pulling between the detector and the corresponding cables. In addition, the alignment quality for optical axes depends on operational experience. A high-quality alignment procedure can be obtained only by an experienced operator and an untrained operation may take more time to implement and may induce manual error.

**Figure 1:**
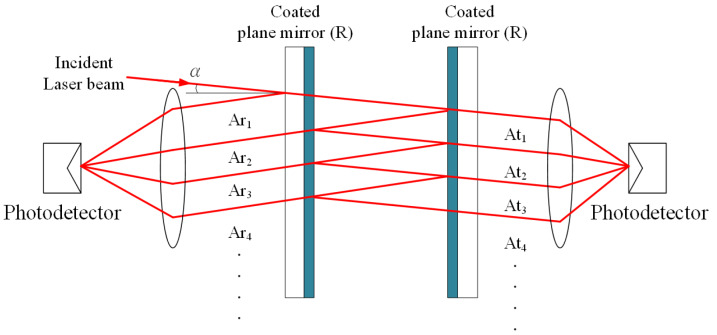
Conventional Fabry–Pérot interferometer.

In accordance with the optical design, laser interferometers can be summarized as two types that contain a non-common path and a common path structure. Commercial laser interferometers are mainly based on the Michelson interferometer with the non-common path structure. The measured displacement is determined by the optical path difference between the reference and the measurement beam [16,17]. Except for the measurement beam, the condition of a reference optical path will also be introduced to the measurement result. In contrast to the Fabry–Pérot interferometer based on a common path structure, there is no additional reference beam in the optical design and laser beams are transmitted under the same optical path [18,19,20]. Therefore, the measured displacement can be precisely defined by its optical cavity and this kind of interferometer has a high resistance of thermal expansion, vibration, and micro-flow gradient [21]. The conventional Fabry–Pérot interferometer (Figure 1) was presented by Charles Fabry and Alfred Pérot in 1897 [22]. The laser beam with a tiny incident angle (α) spreads into the optical cavity composed of two coated plane mirrors. Laser beams are transmitted forward and backward reciprocally and then they are divided into numerous reflected or transmitted beams that are detected by the photodetector. The equation of the light intensity for the interferometric signal is indicated in Equation (1), where A_0_ is the amplitude of the incident beam, R is the reflectance of the plane mirror, respectively.

(1) $$I=\frac{A_{0}{}^{2}\cdot {\left(1-R\right)}^{2}}{1+R^{2}-2\cdot R\cdot \cos\left(\frac{4\pi d}{\lambda }\right)}$$

Due to the reference and measurement beams traveling over the same optical path, the conventional Fabry–Pérot interferometer can be performed for precise length measurement which indicates that phase errors induced by environmental effects can be corrected by this optical arrangement. Nevertheless, the parallelism between two plane mirrors is hard to maintain which leads to measurement limitation over a large range and the signal processing based on the fringe counting method is not capable of determining the moving direction of the tested target. Consequently, this interferometer is rarely employed in dynamic displacement measurements. The quadrature phase-shift fiber-optic Fabry–Pérot interferometer was proposed by Kent A. Murphy et al., in 1999 [23]. The signal processing is according to the spatial phase adjustment to obtain the quadrature interferometric signals. The phase-shift depends on the position of two sensor probes, so the optomechanical arrangement and alignment are essential points in this structure. If further thermal expansion and mechanical vibration appear, the quadrature phase-shift is difficult to maintain. For this reason, the dynamic length measurement within the large measuring range is also hard to realize. The polarized Fabry–Pérot interferometer was presented in our previous study [24,25,26,27,28,29]. By utilizing the polarized phase-shifted method, one octadic-wave plate (λ/8) is introduced into the optical cavity. Hence, the orthogonal phase difference between interferometric signals can be achieved and then the measurement signal can be acquired by photodiodes (PDs). In this structure, signal processing with complex feedback control is adopted in order to avoid signal drift and decline. Furthermore, because each measurement signal possesses its own gain and offset control channels, a data acquisition board with high resolution, sampling rate, and multi-channel is needed. In the whole measurement procedure, the signal processing should be carried out in real-time by the conversion of analog to digital and digital to analog. Therefore, the cost of the systematic construction is raised while the measurement velocity of this structure cannot exceed 4 mm/s.

With respect to the installation of the commercial laser interferometer system, the corresponding optical components should first be arranged and then additional optical adjustment is also required so that the reference and measurement beams can be superimposed for displacement counting after optical alignment. For this reason, there is still room for improvement in convenience of installation.

From the outcome of the above description, a novel calibration system with integration of the multi-beam Fabry–Pérot interferometer and the auto-alignment module is proposed and the correlated features are as follows. A convenient and effective coarse alignment method is proposed to reduce effectively the initial angular deviation between the optical axes and the movement axis and then auto-alignment can be performed within the measuring range of millimeter-scale, not limited by the operational experience and the perceived resolution of the human eye. The entire alignment process can be accomplished in the short-term. Through optimization of the optical design, the linear displacement measurement can be directly implemented after the auto-alignment procedure without extra placement and installation of optical components. For this reason, compact optical arrangement and convenient installation can be realized. The detector for optical alignment is arranged inside the interferometric sensor head which means that cable pulling does not exist in the linear stage. Hence, the risk of optical misalignment and broken cable can be avoided. The signal response also can be enhanced with no existing complex feedback control and advanced data acquisition board by optimization of the signal processing module. Therefore, the proposed system is beneficial for the linear displacement calibration of machine tools in the precision mechanical engineering industry.

## 2. Measurement Principle and Optomechatronic Design

The proposed linear displacement calibration system consists of two chief modules including the linear displacement and the auto-alignment module. Through the optimization and the integration of these two modules, the linear displacement calibration of the linear stage in the machine tool could be realized conveniently and effectively.

### 2.1. Measurement Module for Linear Displacement

**Figure 2:**
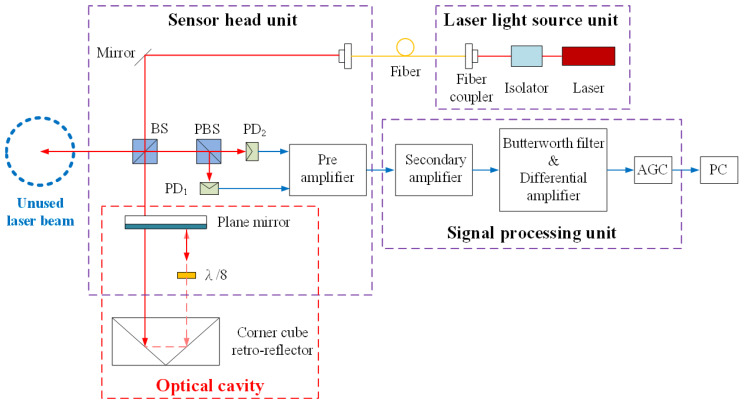
Proposed folded Fabry–Pérot interferometer.

The folded Fabry–Pérot interferometer for determining linear displacement is composed of three measurement units including the laser light source, the sensor head, and the signal processing unit demonstrated in Figure 2. According to this optical structure, the laser beam reflected by the non-polarizing beam-splitter (BS) is not yet employed in other measurements. In order to enhance the measurement function of the proposed system, the unused laser beam will be used for the optical alignment revealed in Section 2.3.

The stabilized He–Ne laser spreads through the isolator, single-mode optical fiber, the BS and into the optical cavity. The optical cavity consists of a plane mirror and a corner cube retro-reflector (CCR) while a λ/8 is arranged in the cavity to produce the orthogonal phase shift between interferometric signals. The backward reflected beams emerging from the optical cavity are reflected by the BS. Afterward, the interference beams are divided by the polarizing beam-splitter (PBS) and obtained by two PDs. According to the proposed structure based on multi-beam interference, the laser beam passes through the optical cavity repeatedly, so the transmittance of the intensity loss from the cavity needs to be considered. In order to obtain the continuous change of the signal, the reflectance of the plane mirror is adjusted for further signal processing [30,31]. The theoretical formula of the interferometric light intensity is illustrated in Equation (2) to Equation (5), where A_0_ is the amplitude of the laser, R and T are the reflectance and transmittance of the plane mirror, L is the transmittance evaluated by the intensity loss in the optical cavity. φ is the phase difference which equals to 8πnd/λ_0_, where λ_0_ is the vacuum wavelength, d is the distance of the optical cavity, and n is the refractive index while m is the order number of the backward reflected laser beam, w is the frequency of the electric field, x is the initial traveling path, and k is the wave number.

The electric field of s-type and p-type can be denoted as Equations (2) and (3).

(2) $$E_{\mathrm{Sn}}=\frac{1}{2\sqrt{2}}A_{0}T{\left(L\right)}^{m-1}{\left(\sqrt{R}\right)}^{2m-3}e^{i\left(\omega t-\mathrm{kx}+\left(2m-2\right)\cdot \varphi \right)}$$

(3) $$E_{\mathrm{Pn}}=\frac{1}{2\sqrt{2}}A_{0}T{\left(L\right)}^{m-1}{\left(\sqrt{R}\right)}^{2m-3}e^{i\left(\omega t-\mathrm{kx}+\left(2m-2\right)\cdot \varphi -\frac{\pi }{2}\right)}$$

**Figure 3:**
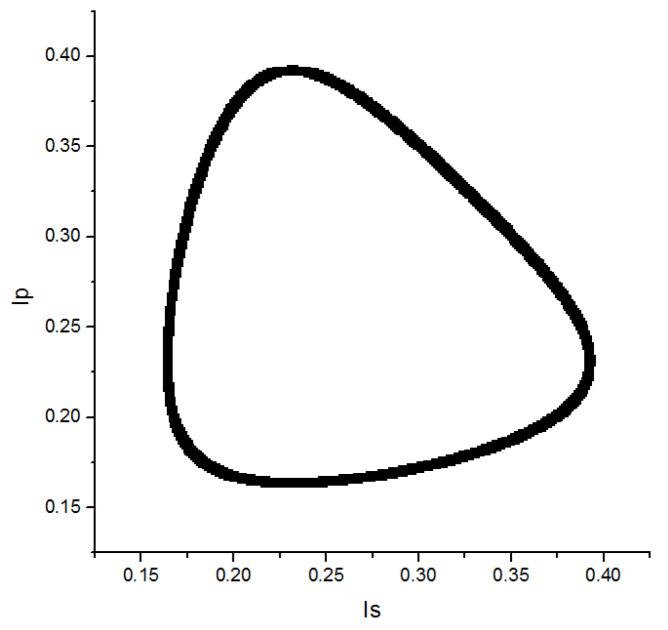
Orthogonal signal of the folded Fabry–Pérot interference.

The intensity distribution of s-type and p-type can be described with Equations (4) and (5). Then the simulation of the orthogonal signal is shown in Figure 3, where A_0_, R, and L are equal to 1, 0.25, and 0.86 respectively. In this theoretical simulation, focus is on observing the curve distribution of the orthogonal interferometric signal and not on analyzing the displacement measurement. Therefore, the cavity refractive index is not considered in the analysis. In the experiment, although the wavelength in the air (λ) is affected by the refractive index (Equation (6)), this refractive index can be corrected by Edlén theoretical formula in Equation (7), where P, T, and RH are atmospheric pressure (kPa), temperature (°C), and relative humidity (%) respectively [32].

(4) $$I_{S}=E_{s}\cdot E_{s}{}^{*}=\frac{1}{8}A_{0}{}^{2}\frac{R\left(1+L^{2}\right)-2\mathrm{LR}\cos\left(2\varphi \right)}{1+L^{2}R^{2}-2\mathrm{LR}\cos\left(2\varphi \right)}$$

(5) $$I_{P}=E_{p}\cdot E_{p}{}^{*}=\frac{1}{8}A_{0}{}^{2}\frac{R\left(1+L^{2}\right)-2\mathrm{LR}\cos\left(2\varphi -\frac{\pi }{2}\right)}{1+L^{2}R^{2}-2\mathrm{LR}\cos\left(2\varphi -\frac{\pi }{2}\right)}$$

(6) $$\lambda =\frac{\lambda_{0}}{n}$$

(7) $$n=1+\frac{7.86\times 10^{-4}\times P}{273+T}-1.5\times 10^{-11}\times \mathrm{RH}\times \left(T^{2}+160\right)$$

The pre-amplifier for two interferometric signals is introduced in the sensor head unit in order to reduce the noise effect, and then signals can be transmitted to the signal processing unit. The purpose is to eliminate the DC offset and to retain the signal amplitude during the measurement process. The signal processing components and procedure are as follows.

The pre-amplifier, secondary amplifier, and differential amplifier are based on the low offset operational amplifier of LF412. The Butterworth filter is adopted and then in the automatic gain control (AGC) circuit, it is mainly integrated with a variable gain amplifier VCA810 and a high-speed comparator AD8561. The elimination of the DC offset can be obtained by further signal processing with a Butterworth filter and a differential amplifier. In the AGC circuit, the gain amplifier VCA810 relies on the control voltage obtained by feedback to control the magnification. The comparator AD8561 is utilized to compare the output signal with the setup voltage. After passing through the detection circuit, amplification can be performed by the gain amplifier VCA810. Therefore, the signal magnification can be auto-adjusted in accordance with the output signal, so that the signal amplitude remains almost the same during the displacement measurement. Compared to previous signal processing, the need of the elimination of the DC offset and the retention of the signal amplitude can be realized only by a correlated circuit without complex feedback control and an advanced multi-channel data acquisition board. Therefore, the signal response can be enhanced according to the optimized signal processing.

### 2.2. Auto-Alignment Module for Optical Axes

The alignment procedure including coarse alignment and fine alignment is to align the optical axes of the laser interferometer and the movement axis of the linear stage to a parallel state. After coarse alignment, the initial angular deviation between the optical axes of the interferometer and the movement axis of the linear stage can be reduced and then the laser spot will be at the sensing area of the detector within the whole alignment distance. This indicates that the fine alignment can be performed automatically.

**Figure 4:**
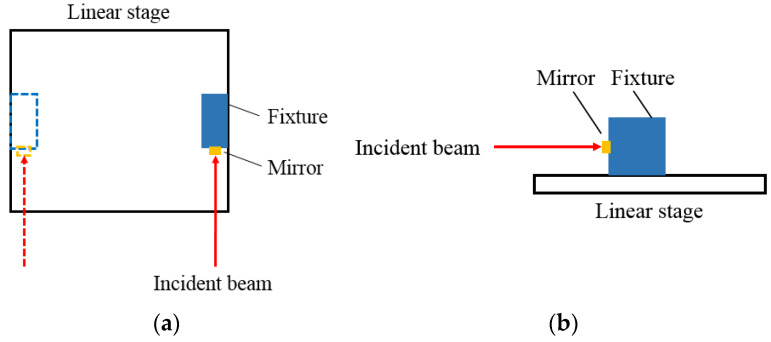
Coarse alignment: (**a**) top view and (**b**) side view.

For CNC machine tools, geometric errors e.g., the straightness of a single axis, and the parallelism and squareness between multi-axes, should be detected before delivery. If large geometric errors occur, they must be corrected and minimized during the assembly process. In view of the above-mentioned description, a compact fixture with high squareness could be utilized as a reference for the two angular directions (pitch, yaw) of the linear stage. In the coarse alignment procedure, the fixture with a mirror is placed in the linear stage and aligned to one side of it, as shown in Figure 4. Then the laser beam from the sensor head is incident to this mirror, meeting the orthogonal incident situation between the integrated fixture and the laser beam. Hence, the initial angular deviation between the optical axes and the movement axis can be easily and quickly reduced.

**Figure 5:**
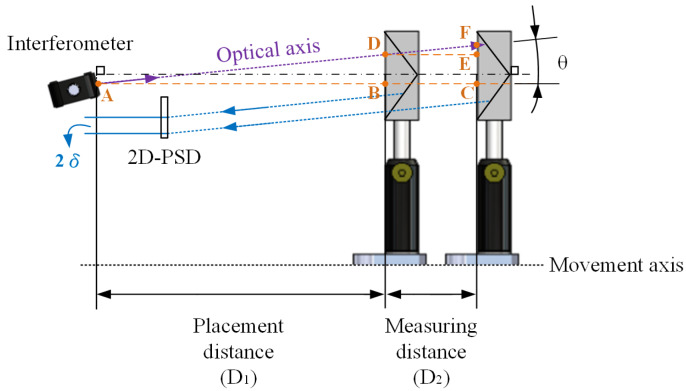
Fine alignment.

When the coarse alignment procedure is accomplished, a CCR is placed on the linear stage and then the laser beam incident to it, will be retro-reflected into the two-dimensional PSD (2D-PSD) fixed in the sensor head to carry out the fine alignment procedure illustrated in Figure 5.

When there is an angular deviation between the optical axes and the movement axis over the whole measuring distance, a triangular geometric relationship can be obtained through their corresponding positions. It contains ΔACF and ΔDEF, where $\bar{\mathrm{AB}}$ is the placement distance of the sensor head (D_1_) and $\bar{\mathrm{BC}}$ is the measuring distance (D_2_). From Equations (8) and (9), the fine aligned distance ($\bar{\mathrm{CF}}$) and the tilt angle (θ) between two axes can be acquired, respectively.

(8) $$\bar{\mathrm{CF}}=\frac{\bar{\mathrm{AC}}\;\times \;\bar{\mathrm{EF}}}{\bar{\mathrm{DE}}}=\frac{\left(D1+D2\right)\;\times \;\delta }{D2}$$

(9) $$\theta =\tan^{-1}(\frac{\bar{\mathrm{CF}}}{\bar{\mathrm{AC}}})$$

**Figure 6:**
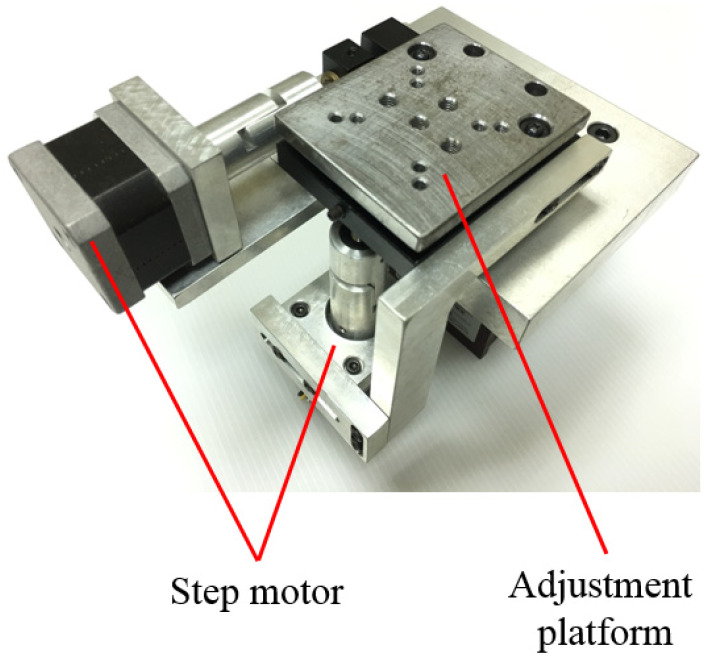
Mechanism design for auto-alignment.

The mechanism design for auto-alignment, composed of two step motors and an adjustment platform, is shown in Figure 6. The laser interferometer is fixed on the adjustment platform and then the two rotary axes of it are adjusted by two motors, respectively. The cosine error can be determined by the pitch and yaw angle during the alignment process.

### 2.3. Linear Displacement Calibration System

**Figure 7:**
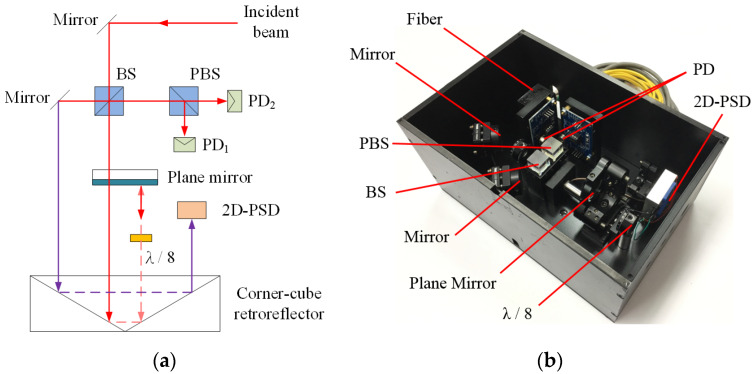
Proposed linear displacement calibration system: (**a**) Optical structure and (**b**) optomechanical structure.

By integrating two modules, including a measurement module for linear displacement and an auto- alignment module for the optical axes, a linear displacement calibration system was proposed. The unused laser beam revealed in Section 2.1 was employed to align the optical axes by further optical arrangement. Then the two major laser beams which travel along the inner and outer edge of the CCR can be utilized to measure the linear displacement (red line) and align the optical axes (purple line) respectively, shown in Figure 7. Namely, the need of optical alignment and linear displacement measurement can be achieved simultaneously. Therefore, by the proposed novel optical design, laser beams are fully arranged to improve the usability and measurement function of the proposed system and optical misalignment and broken cable will not occur. Moreover, further straightness measurements could also be implemented in this structure. 

## 3. System Structure

**Figure 8:**
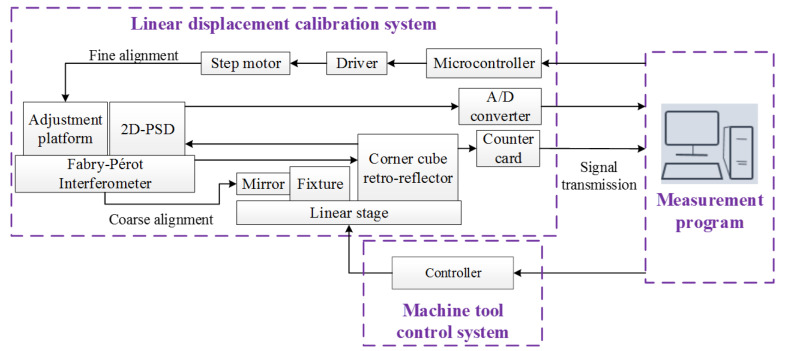
System structure.

The system structure for the linear displacement calibration consists of a linear displacement calibration system, a machine tool control system, and a measurement program, as demonstrated in Figure 8. A high stabilized He–Ne Laser source with awavelength of about 632.8 nm and output power of about 1.5 mw is utilized in the linear displacement calibration system. Before the calibration procedure, a fixture with squareness of about 0.1 mm is arranged in the linear stage to carry out coarse alignment. Following this, the laser spot incident to CCR is retro-reflected to the 2D-PSD and the signal is processed by an A/D converter whose sampling rate and resolution are 1 MHz and 16-bit respectively. The pitch and yaw angle of the interferometric sensor head is adjusted by the step motors with a resolution of about 13 arc-seconds. When the optical alignment procedure is accomplished, the linear displacement calibration of the machine tool is performed according to the international specification ISO 230-2 to set measurement times and intervals [33]. Then the interferometric signal is conducted through the signal processing unit and counted by the counter card whose sampling rate is 10 MHz. The corresponding specifications of the signal processing unit are as follows; the gain of pre-amplifier and secondary amplifier is over a range from 6 to 20 dB, the response of Butterworth filter rolls off at −6 dB per octave, and in the AGC circuit, the adjusted gain ranges from −40 to 40 dB while the gain fluctuation is less than 1 dB in the frequency range from 300 Hz to 30 MHz.

## 4. Results and Analysis

### 4.1. Auto-Alignment for Optical Axes

#### 4.1.1. Calibration Test of 2D-PSD

**Figure 9:**
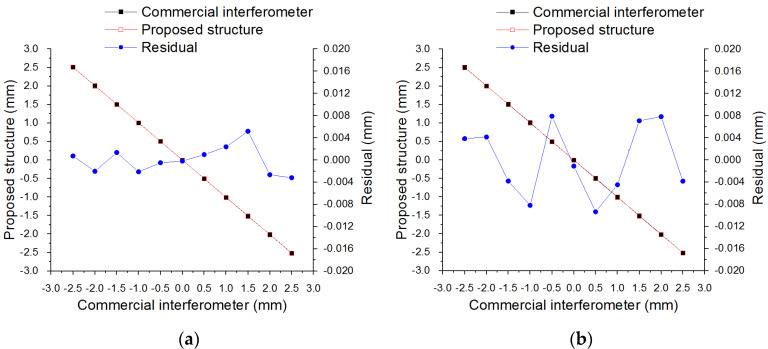
Calibration test of two-dimensional PSD (2D-PSD) in two directions: (**a**) X direction and (**b**) Y direction.

In the calibration test, the lateral offset is regulated from −2.5 mm to 2.5 mm with a step interval of 0.5 mm and it is obtained by the proposed structure and the commercial laser interferometer at each position. After the calibration, characteristic curves illustrated in Figure 9 reveal that the maximum residuals of the X direction and the Y direction are 5 μm and −9 μm respectively within the range of ±2.5 mm. Therefore, optical alignment can be carried out according to the performance of the proposed structure.

#### 4.1.2. Optical Alignment

The fixture with squareness of about 0.1 mm is placed on one side of the linear stage and then the laser beam from the interferometric sensor head should be incident to the mirror orthogonally. In the experiment, the 2D-PSD whose sensing area is 10 × 10 mm^2^ was utilized to acquire the position of the laser spot at the start-point and endpoint of the tested range of 100 mm to verify the angular deviation after coarse alignment. The results shown in Table 1 reveal that the angular deviation is less than 0.226 degrees which equals a deviation of about 0.986 mm within a distance of 250 mm. From the results of the coarse alignment, the angular deviation between the optical axes and the movement axis can be reduced effectively and then the auto-alignment can be carried out in the measuring range of a millimeter-scale. On assuming the employment of 70% sensing area of the detector, the auto-alignment process can be performed over the whole distance of about 440 mm. If a more precise commercial fixture with squareness of about 0.002 mm in a length of 150 mm is considered, the corresponding deviation will be reduced to about 0.605 mm within the same distance and the available range for auto-alignment will be improved by a factor of about 1.6.

After coarse alignment, the laser spot is within the sensing area of the 2D-PSD over the whole measuring distance of 200 mm, and then fine alignment is executed automatically. Five experiments are performed and the deviation of the X and Y directions is obtained in order to estimate the existing cosine error over the whole scale after this procedure. The experimental result indicated that the cosine error could be reduced to less than 0.36 nm and the averaged fine alignment time was 45 s (Table 2). The fine alignment does not involve any detector and corresponding cables at the linear stage in order to reduce the risk of misalignment and broken cable. The whole fine alignment process is performed automatically and does not depend on operational experience. The entire optical alignment procedure can be accomplished within 75 s. Furthermore, the linear displacement measurement can be performed directly after the optical alignment with no additional installation and adjustment. For this reason, a compact structure and convenient employment can be achieved while the inspection efficiency is enhanced by 80%.

### 4.2. Comparison Experiment of Linear Displacement

**Figure 10:**
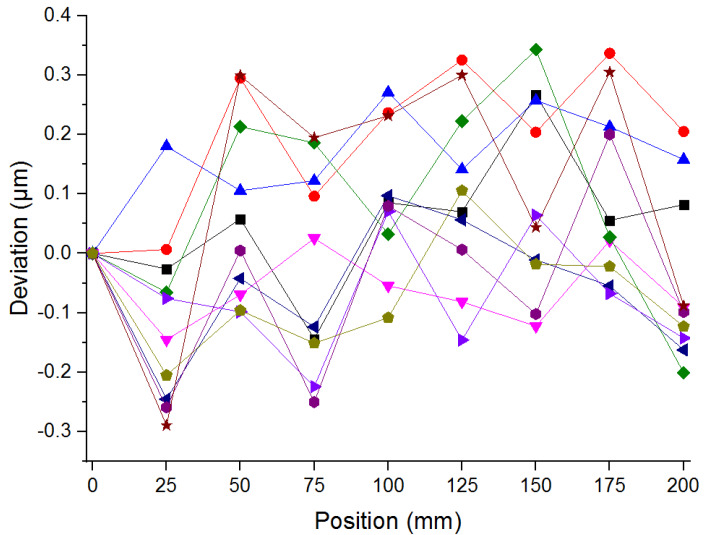
Comparison result of linear displacement.

In order to evaluate the entire measurement performance of the proposed system, the maximum standard deviation was analyzed in this study. A comparison experiment between the self- developed structure with a resolution of λ/16 and the commercial interferometer with a resolution of 1 nm was carried out. Forward displacement experiments were repeated ten times in the measuring range of 200 mm, conducted with a step interval of 25 mm. The comparison result between both systems demonstrated in Figure 10 reveals that the maximum deviation is 0.343 μm and the maximum standard deviation is 0.171 μm. 

The measured standard deviation was compared to the results of the previous study to verify the feasibility of the proposed measurement module for linear displacement. In the measuring range of millimeter-scale, the comparison results shown in Table 3 reveal that the maximum standard deviation of the previous study is less than 0.260 μm and that of the proposed system is less than 0.171 μm. Both are all of the same order of the sub-micrometer. Therefore, the precise measurement performance of the proposed system could be verified. This performance should be available for precise displacement measurements to meet the demand of the precision mechanical engineering industry.

The wavelength in the air is affected by the corresponding refractive index and this index is also a function of temperature which is the major influence factor on the laser wavelength during the linear displacement measurement. This error caused by a tiny temperature difference can be corrected by the Edlén theoretical formula (Equation (7)). The temperature value obtained in the measurement procedure is substituted in this formula to carry out wavelength compensation and then the refractive index can be corrected. Due to the measurement procedure performed in the short- term (<12 min) and the sensor head possessing an isolated protective cover, the temperature fluctuation was lower (<±0.2 °C). According to JCGM100, the uncertainty was evaluated which equals about ±23.1 nm in the measuring range of 200 mm.

### 4.3. Linear Displacement Calibration of the Machine Tool

**Figure 11:**
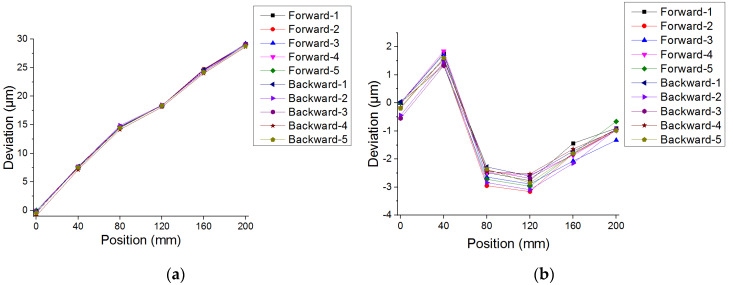
Linear displacement calibration of the machine tool: (**a**) Without compensation and (**b**) with compensation.

For linear displacement calibration, there are six measurement points in the whole stroke of 200 mm and each procedure is repeated five times. Experimental results of the linear positioning error in the forward and backward direction performed by the proposed system are shown in Figure 11.

The systematic positioning deviation and the repeatability and accuracy of the machine tool are determined in accordance with the international specification of ISO230-2. The calibration result for the linear axis of the machine tool is illustrated in Table 4. With the compensation procedure, it is revealed that the systematic positional deviation, repeatability, and accuracy are 4 μm, 1 μm, and 5 μm respectively. Therefore, the demands of the linear displacement measurement and the optical alignment can be met simultaneously through the extension and the optimization of the proposed system which is robust and efficient for the calibration of the linear stage in the machine tool.

## 5. Conclusions

In this study, a linear displacement calibration system integrated with a linear displacement measurement module based on the Fabry–Pérot interferometer and an auto-alignment module was proposed for the automatic calibration of the linear stage in a machine tool. After optical alignment, the cosine error could be improved to 0.36 nm and the entire procedure accomplished within 75 s. In the comparison results of linear displacement, the standard deviation was less than 0.171 μm in the measuring range of 200 mm. According to the linear displacement calibration results, the systematic positional deviation, repeatability, and accuracy of the linear axis could be improved to 4 μm, 1 μm, and 5 μm respectively. Therefore, the proposed precise structure possesses a compact characteristic and can be performed for the calibration of machine tools conveniently and effectively. In future work, a straightness measurement will be implemented in this structure simultaneously and the automatic compensation module of the linear positioning error will also be integrated to realize a fully automated calibration system for automatic alignment, measurement. and compensation.

## Figures and Tables

**Table 1:** Results of coarse alignment.

Times	1		2		3	
Direction	X	Y	X	Y	X	Y
Deviation (mm)	−0.395	0.335	−0.193	0.328	0.305	0.344
Angle (degree)	−0.226	0.192	−0.110	0.188	0.175	0.197
Alignment time (s)	30					

**Table 2:** Results of fine alignment.

Times	1		2		3		4		5	
Direction	X	Y	X	Y	X	Y	X	Y	X	Y
Deviation (μm)	−3	−6	9	11	10	9	−8	12	−4	7
Angle (degree × 10^−3^)	−0.9	−1.7	2.6	3.2	2.9	2.6	−2.3	3.4	−1.1	2.0
Cosine error (nm)	0.02	0.09	0.20	0.30	0.25	0.20	0.16	0.36	0.04	0.12
Average alignment time (s)	45									

**Table 3:** Comparison results of the proposed system.

Item		Resolution (nm)	Dynamic Range (mm)	Repeatability (μm)
Previous system/Reference number	[24]	40	160	0.255
	[25]	2.5	500	0.146
	[26]	40	100	0.211
Proposed system		40	200	0.171

**Table 4:** Calibration results of the linear axis.

Parameters (μm)	Without Compensation	With Compensation
Systematic positional deviation (E)	30	4
Repeatability (R)	1	1
Accuracy (A)	30	5

## References

[1] Jaeger G. (2010). Three-Dimensional Nanopositioning, Nanomeasuring Machine with a Resolution of 0.1 nm. Optoelectronics. Instrum. Data Process..

[2] Pozzo W.D. (2011). Inference of cosmological parameters from gravitational waves: Applications to second generation interferometers. Phys. Rev. D Part. Fields.

[3] Raina G. (2013). Atomic Force Microscopy as a Nanometrology Tool: Some Issues and Future Targets. J. Metrol. Soc. India.

[4] Lee J.C., Lee K.I., Yang S.H. (2016). Development of compact three-degrees-of-freedom compensation system for geometric errors of an ultra-precision linear axis. Mech Mach Theory.

[5] Ramesh R., Mannan M.A., Poo A.N. (2000). Error compensation in machine tools — a review Part I: Geometric, cutting-force induced and fixture-dependent errors. Int. J. Mach. Tools Manuf..

[6] Viprey F., Nouira H., Lavernhe S., Tournier C. (2016). Novel multi-feature bar design for machine tools geometric errors identification. Precis. Eng..

[7] Tana K.K., Huang S.N., Lee T.H. (2003). Dynamic S-function for geometrical error compensation based on neural network approximations. Measurement.

[8] Castro H.F.F., Burdekin M. (2003). Dynamic calibration of the positioning accuracy of machine tools and coordinate measuing machines using a laser interferometer. Int. J. Mach. Tools Manuf..

[9] Renishaw XL-80 Laser Measurement System. https://www.renishaw.com/media/pdf/en/5d15dd21874642ba986dbdefb6ede174.pdf.

[10] Keysight Technologies 5530 Dynamic Calibrator. https://www.keysight.com/main/redirector.jspx?action=ref&cname=EDITORIAL&ckey=1750511&c=cht&cc=TW&nfr=-536900386.0.00.

[11] API XD LASER. https://apimetrology.com/machine-tool-brochure-form/213.

[12] Renishaw XM-60 Multi-Axis Calibrator. http://resources.renishaw.com/en/details/brochure-xm-60-multi-axis-calibrator--111830.

[13] Huang Q., Liu X., Sun L. Homodyne laser interferometric displacement measuring system with nanometer accuracy. Proceedings of the Ninth International Conference on Electronic Measurement & Instruments.

[14] Jywe W.Y., Hsieh T.H., Chen P.Y., Wang M.S. (2018). An Online Simultaneous Measurement of the Dual-Axis Straightness Error for Machine Tools. Appl. Sci..

[15] Hsieh T.H., Chen P.Y., Jywe W.Y., Chen G.W., Wang M.S. (2019). A Geometric Error Measurement System for Linear Guideway Assembly and Calibration. Appl. Sci..

[16] Gregorčič P., Tomaž P., Janez M. (2009). Quadrature phase-shift error analysis using a homodyne laser interferometer. Opt. Express.

[17] Greco V., Molesini G., Quercioli F. (1995). Accurate polarization interferometer. Rev. Sci. Instrum..

[18] Rabinowitz P., Jacobs S.F., Shultz T., Gould G. (1962). Cube-corner Fabry-Perot interferometer. J. Opt. Soc. Am..

[19] Pietraszewski K.A.R.B. (2000). Recent Developments in Fabry-Perot Interferometer. ASP Conference Series.

[20] Lawall J.R. (2005). Fabry-Perot metrology for displacements up to 50 mm. J. Opt. Soc. Am. A Opt. Image. Sci. Vis..

[21] Wang Y.C., Shyu L.H., Chang C.P. (2010). The Comparison of Environmental Effects on Michelson and Fabry-Perot Interferometers Utilized for the Displacement Measurement. Sensors.

[22] Vaughan J.M. (1989). Fabry–Perot Interferometer History Theory Practice and Applications.

[23] Murphy K.A., Gunther M.F., Vengsarkar A.M., Claus R.O. (1991). Quadrature phase-shifted, extrinsic Fabry–Perot optical fiber sensors. Opt. Lett..

[24] Shyu L.H., Wang Y.C., Chang C.P., Tung P.C., Manske E. (2012). Investigation on displacement measurements in the large measuring range by utilizing multibeam interference. Sens. Lett..

[25] Chang C.P., Tung P.C., Shyu L.H., Wang Y.C., Manske E. (2013). Modified Fabry-Perot Interferometer for Displacement Measurement in Ultra Large Measuring Range. Rev. Sci. Instrum..

[26] Chang C.P., Tung P.C., Shyu L.H., Wang Y.C., Manske E. (2013). Fabry–Perot displacement interferometer for the measuring range up to 100 mm. Measurement.

[27] Shih H.T., Wang Y.C., Shyu L.H., Tung P.C., Chang C.P., Jywe W.Y., Chen J.H. (2018). Automatic calibration system for micro-displacement devices, Measurement Science and Technology. Meas. Sci. Technol..

[28] Wang Y.C., Shyu L.H., Chang C.P. (2010). Fabry–Pérot interferometer utilized for displacement measurement in a large measuring range. Rev. Sci. Instrum..

[29] Shyu L.H., Wang Y.C., Chang C.P., Shih H.T., Manske E. (2016). A signal interpolation method for fabry-perot interferometer utilized in mechanical vibration measurement. Measurement.

[30] Shyu L.H., Chang C.P., Wang Y.C. (2011). Influence of Intensity Loss in the Cavity of a Folded Fabry-Perot Interferometer on Interferometric Signals. Rev. Sci. Instrum..

[31] Wang Y.C., Shyu L.H., Tung P.C., Shih H.T., Lin J.C., Lee B.Y., Li J.S. (2017). Optimization of the optical parameters in Fabry-Perot interferometer. Ilmenau Sci. Colloq..

[32] Edlen B. (1966). The Refractive Index of Air. Metrologia.

[33] (1997). Test Code for Machine Tools–Part 2: Determination of Accuracy and Repeatability of Positioning of Numerically Controlled Axes.

